# A software module to assess the metabolic potential of mutant strains of the bacterium Corynebacterium glutamicum

**DOI:** 10.18699/vjgb-24-97

**Published:** 2024-12

**Authors:** F.V. Kazantsev, M.F. Trofimova, T.M. Khlebodarova, Yu.G. Matushkin, S.A. Lashin

**Affiliations:** Kurchatov Genomic Center of ICG SB RAS, Novosibirsk, Russia Institute of Cytology and Genetics of the Siberian Branch of the Russian Academy of Sciences, Novosibirsk, Russia Novosibirsk State University, Novosibirsk, Russia; Institute of Cytology and Genetics of the Siberian Branch of the Russian Academy of Sciences, Novosibirsk, Russia; Kurchatov Genomic Center of ICG SB RAS, Novosibirsk, Russia Institute of Cytology and Genetics of the Siberian Branch of the Russian Academy of Sciences, Novosibirsk, Russia; Kurchatov Genomic Center of ICG SB RAS, Novosibirsk, Russia Institute of Cytology and Genetics of the Siberian Branch of the Russian Academy of Sciences, Novosibirsk, Russia Novosibirsk State University, Novosibirsk, Russia; Kurchatov Genomic Center of ICG SB RAS, Novosibirsk, Russia Institute of Cytology and Genetics of the Siberian Branch of the Russian Academy of Sciences, Novosibirsk, Russia Novosibirsk State University, Novosibirsk, Russia

**Keywords:** flux models, bacterial metabolism, metabolic optimization, rational metabolic engineering, потоковые модели, метаболизм бактерии, оптимизация метаболизма, рациональная метаболическая инженерия

## Abstract

Technologies for the production of a range of compounds using microorganisms are becoming increasingly popular in industry. The creation of highly productive strains whose metabolism is aimed to the synthesis of a specific desired product is impossible without complex directed modifications of the genome using mathematical and computer modeling methods. One of the bacterial species actively used in biotechnological production is Corynebacterium glutamicum. There are already 5 whole-genome flux balance models for it, which can be used for metabolism research and optimization tasks. The paper presents fluxMicrobiotech, a software module developed at the Institute of Cytology and Genetics of the Siberian Branch of the Russian Academy of Sciences, which implements a series of computational protocols designed for high-performance computer analysis of C. glutamicum whole-genome flux balance models. The tool is based on libraries from the opencobra community (https://opencobra.github.io) within the Python programming language (https://www.python.org), using the Pandas (https://pandas.pydata.org) and Escher (https://escher.readthedocs.io) libraries . It is configured to operate on a ‘file-in/file-out’ basis. The model, environmental conditions, and model constraints are specified as separate text table files, which allows one to prepare a series of files for each section, creating databases of available test scenarios for variations of the model. Or vice versa, allowing a single model to be tested under a series of different cultivation conditions. Post-processing tools for modeling data are set up, providing visualization of summary charts and metabolic maps.

## Abstract (ru)

Технологии производства различных соединений с применением микроорганизмов приобретают все большую популярность в промышленном производстве. Создание современных высокопродуктивных штаммов, метаболизм которых ориентирован на синтез конкретного целевого продукта, невозможно без комплексной направленной модификации генома c применением методов математического и компьютерного моделирования. Одним из видов бактерий, активно используемых в биотехнологическом производстве, является Corynebacterium glutamicum. Для него существует уже пять полногеномных потоковых моделей, которые можно использовать для задач исследования и оптимизации метаболизма. В работе представлен программный модуль развиваемого в Институте цитологии и генетики СО РАН инструмента FluxMicrobiotech, в рамках которого реализована серия вычислительных протоколов, предназначенных для массового компьютерного анализа потоковых моделей C. glutamicum на высокопроизводительных вычислительных компьютерах. Программный модуль реализован на языке Python с применением библиотек Pandas, cobraPy и Escher и настроен на работу по принципу «файл на вход/файл на выход». Модель, условия среды и ограничения модели задаются как отдельные текстовые табличные файлы, что позволяет заготовить серию файлов для каждого из разделов, создавая базы доступных сценариев испытаний для вариаций модели. Или, наоборот, позволяет испытывать одну модель в серии разных условий культивирования. Настроены инструменты постобработки данных моделирования, обеспечивающие визуализацию сводных диаграмм и метаболических карт.

## Introduction

Technologies for the production of a range of compounds using microorganisms are becoming increasingly popular in the industry. Creation of modern highly productive microorganism strains, the metabolism of which is focused on synthesis of a specific target product, is impossible without complex directed genome modifications. To date, a wide range of rational and systemic metabolic engineering methods have been developed to increase the production of target substances (Sheremetieva et al., 2023, 2024), the use of which, together with computer modelling approaches, will make it possible to more accurately assess the impact of genome changes on the dynamics of the system and the yield of the final product (Ananda et al., 2024). Implementation of the flux-based mathematical modelling methods for molecular genetic and metabolic systems within the computational modelling frameworks (Mendoza et al., 2019; Mao et al., 2023) and creation of whole-genome flux-based mathematical models allow in silico prediction of genetic modifications required to increase culture growth rate and target product yield under optimal conditions on different substrates (Gu et al., 2019; Mao et al., 2023).

One of the bacterial species actively used in biotechnological production is Corynebacterium glutamicum. Since its discovery in 1956 (Kinoshita et al., 1957) until now, the main application of this bacterial species has been the production of amino acids and their derivatives (Tsuge, Matsuzawa, 2021), which is currently the second most economically important process in industrial biotechnology (Barcelos et al., 2018). C. glutamicum are non-pathogenic, GC-rich, Gram-positive soil bacteria. They do not form spores, grow rapidly, do not require special conditions for growth, do not secrete proteases, have a relatively stable genome and are resistant to high concentrations of potentially toxic substances, making this microorganism an ideal platform for the development of industrially relevant strains based on it (Wendisch et al., 2016).

The main approaches for modifying the genome of biotechnologically relevant bacterial strains include: 1) gene knockouts (switching off); 2) insertion of additional genes leading to the creation of new metabolic reaction chains; 3) insertion of mutations both in the regulatory regions of genes and in the structure of genes in order to decrease/increase gene expression and activity of their products, respectively; 4) other modern methods of C. glutamicum genome editing, without which it is impossible to realize a large number of directed modifications necessary for the implementation of rational and systemic metabolic engineering approaches (Sheremetieva et al., 2023, 2024). Effective planning, execution and control of such modifications are difficult without the use of mathematical and computational modelling techniques

The paper is dedicated to the development of a software module within the framework of the FluxMicrobiotech toolkit created at the Institute of Cytology and Genetics SB RAS. The toolkit was created to assess the metabolic potential of a bacterium using flux modelling methods, including a set of computational protocols configured for massive computational analysis of the metabolism of target bacterial strains when cultivated on different nutrient media and under different environmental conditions (aerobic/anaerobic)

## Materials and methods

The developed computational protocols are based on the open source flux modelling methods library opencobra (opencobra. github.io) within the Python programming language (https:// www.python.org/). The protocols are designed as “notebooks” in the Jupyter programming environment (https://jupyter. org/). This structure allows combining computational blocks with stages of results analysis. The approach of organizing computations using “notebooks” has become a familiar tool in big data analysis methodology, implying the creation of computational pipelines and their regular adjustment to changing objective conditions. Control of the correct use is gained by a powerful toolkit of annotations to the calculation stage. The cobraPy (https://opencobra.github.io/cobrapy/) and Pandas (https://pandas.pydata.org/) libraries are used to solve optimization problems. The yEd Graph Editor (https:// yworks.com/products/yed) is used for the raw visualization of gene networks. Creation of metabolic maps and plotting of solutions on them during modelling is implemented in the Escher toolkit (escher.github.io/). The developed protocols support high-performance computing methods and require memory to store the results. Thus, it is recommended to carry out the work on high-performance computers

The flow modelling techniques (the alternative term is FBA – Flux Balance Analysis) used in this paper belong to the linear programming problem domain. It is to address the challenges of metabolic research that a series of computational FBA| method libraries are being developed within the opencobra community (https://opencobra.github.io). The basis of this methodology is the representation of the metabolic pathway as a graph given by an adjacency matrix with the rows corresponding to metabolites, and the columns, to metabolic reactions and processes. Matrix elements are stoichiometric coefficients specifying the proportion of a metabolite and its role in the selected reaction (reagent or reaction product). Such matrices can be constructed manually by carefully describing the target metabolic pathways, or automatically by generating a matrix from genomic information. Using a well-annotated bacterial genome sequence and various bioinformatics tools, potential metabolic pathways and the bacterium’s ability to synthesize target metabolites can be identified. It is this information that is processed by software tools for generating Whole Genome Flux Models (the alternative term is GSM – genome-scale metabolic models) (Machado et al., 2018; Kulyashov et al., 2023).

A flux model constructed in the manner mentioned above is a starting point in the task of assessing the metabolism of a bacterium and can contain several thousand reactions describing the full set of functionalities available in the genome. There is the BiGG database (http://bigg.ucsd.edu/), which is positioned as a central point for storing and reusing flux models. This resource contains the largest collection of whole-genome mathematical models developed for different organisms, and in addition is being developed as a database of reference biochemical reactions for these types of models as well. Within BiGG, the Escher metabolic network visualization tool (King et al., 2015) is being developed in parallel, allowing the same metabolic maps to be reused for models of different organisms. The BiGG database contains 108 published and manually validated whole-genome metabolic models for 40 different organisms (Norsigian et al., 2019).

**Fig. 1.**
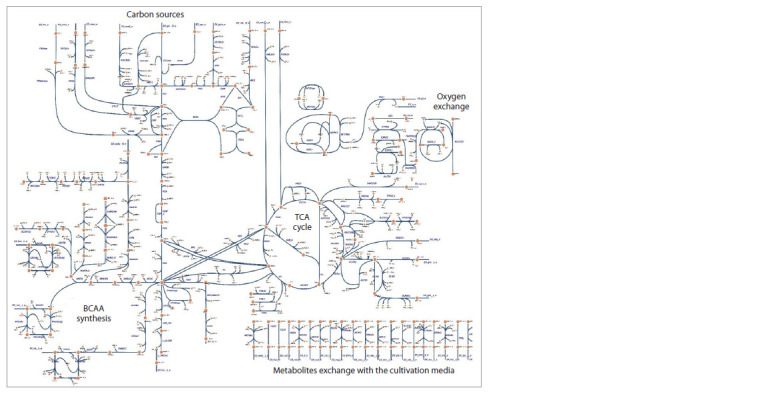
Metabolic map focused on metabolic pathways for the synthesis of branched-chain amino acids (BCAAs). The visualization was done in the Escher tool as an extended network of the iCGB21FR model.

Thus, the bundling of genome data, tools for building and annotating whole-genome flux models, and their integration within the BiGG approach provide the basis for high-throughput computational analyses of bacterial metabolism. While the model is whole-genome, only a subset of the metabolic pathway reactions for key metabolites are used when displaying the metabolic map as a graph (Fig. 1), assuming that pathways not included in the visualization are also involved in the analysis.

## Results

Flux model of Corynebacterium glutamicum

To date, several mathematical models describing the metabolism of the bacterium C. glutamicum have been created and published: iEZ482, iCW773, iCGB21FR, ecCGL1, iJM658 (Kjeldsen, Nielsen, 2009; Zelle et al., 2015; Mei et al., 2016; Zhang et al., 2017; Feierabend et al., 2021; Niu et al., 2022). These models are based on whole-genome data and have been verified on experimental data on bacterial growth, ability to synthesize amino acids on different carbon sources and under different cultivation medium conditions. The models were used to analyse the production of glutamate (Mei et al., 2016; Feierabend et al., 2021), isoleucine (Zhang et al., 2017) and lysine (Kjeldsen, Nielsen, 2009; Zhang et al., 2017; Niu et al., 2022).

**Fig. 2.**
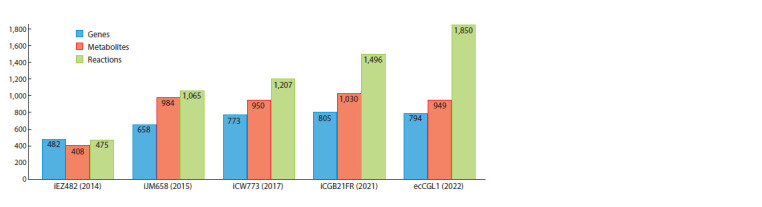
Mathematical models of C. glutamicum metabolism and their main characteristics.

The iEZ482 model was presented in 2015 and describes the metabolism of strain ATCC 13032. It contains 475 metabolic reactions and 408 metabolites. The model was validated by the authors using experimental data on the ability to excrete 20 amino acids. The iCW773 model published in 2017 contains 1,207 reactions and 950 metabolites. Based on iCW773, the ecCGL1 model was published in 2022. It provides a mathematical description of the metabolism of the bacterium C. glutamicum strain ATCC 13032 with enzymatic constraints, in which not only metabolites and reactions are specified, but also constraints on the maximum concentration of enzymes in the bacterium are incorporated. The iJM658 model was built for strain S9114, published in 2016, and contains 658 genes, 984 metabolites and 1,065 reactions. Further development of whole-genome modelling for C. glutamicum ATCC 13032 led to the iCGB21FR model, released in 2021. The model contains 1,496 reactions, 1,030 metabolites, 805 genes and 3 compartments: extracellular space, cytosol and periplasm. Validation of the model was performed by the authors on the metabolism of L-glutamate, which in turn is a precursor for the synthesis of a series of amino acids. Characteristics of the found models are presented in Figure 2.

The iCGB21FR model was chosen as the base model for setting up computational protocols, building metabolic maps and data post-processing tools, as it describes the metabolism of C. glutamicum bacteria in the most complete and up-to-date way. It can also serve as a benchmark for model annotation, as it covers most of the recommendation points in the systems biology model design standard, including references to existing databases and ontologies. The iCGB21FR model is freely available in the BioModels database (https://www.ebi. ac.uk/biomodels, model identifier MODEL2102050001). The model demonstrates the ability of the bacterium to grow on different carbon sources under aerobic and anaerobic conditions on three different culture media: minimal M9 medium, minimal CGXII medium, and complete lysogenic broth (LB) medium. These conditions differ in the quantity and quality (availability of additional carbon or amino acid sources) of metabolites that the model can consume from the culture medium for processing into metabolic products

Computational protocols

**Fig. 3.**
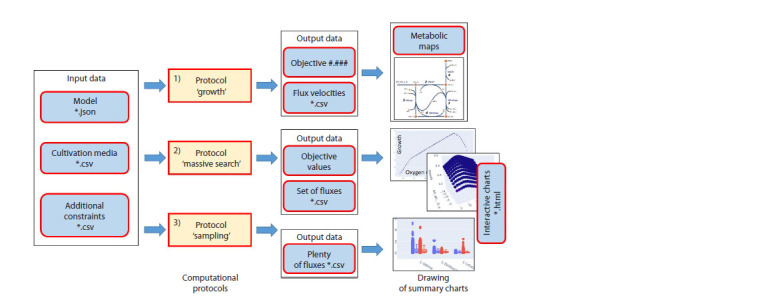
A data flow diagram of computational protocols.

The developed software module contains a series of basic computational scripts, the data flow of which is schematically represented in Figure 3. This is a prepared Jupyter lab notebook in which the calculation parameters are set.

The starting conditions for all protocols are the same:

1) it is necessary to specify the flux model (*.json file), which describes the basic structure and constraints of the model. This model can be obtained from the BIGG databases or created using the cobraPy software toolkit;

2) set the cultivation medium parameters as a tabular text file (*.csv);

3) set additional constraints on model fluxes as a tabular text file (*.csv).

Then, depending on the task to be solved, the calculation parameters are set up. Jupyter lab notebook as a computational protocol allows users to quickly modify each block of calculations if necessary. As a result, the computational protocol is actually specified through a set of files: model, cultivation medium, additional constraints. This provides the ability to prepare a series of files for each section, creating databases of available test scenarios for variations of a model or, conversely, testing a single model under a series of different cultivation conditions.

The result of the protocol is the vector of resulting velocities over the entire model structure (or a set of such vectors in the form of a rectangular matrix). For post-processing tasks, a toolkit has been set up to display data both as result diagrams and as a visualization of flows on a metabolic map (Fig. 1). The task of exporting the results as a series of interactive metabolic maps was done using the Escher toolkit (https:// escher.readthedocs.io).

Scenario for estimating biomass growth

The bacterial cultivation medium plays a major role in biotechnological production. The media can be of minimal biochemical composition or rich in amino acids, so that the bacterium can consume them from the medium rather than spending internal resources to synthesize amino acids and other metabolites. In order to estimate metabolic parameters of strains using modelling, it is necessary to set the cultivation conditions as precisely as possible.

The first test of model adequacy is its ability to predict biomass growth on given substrates in accordance with experimental data. This parameter is usually not difficult to investigate experimentally: there is plenty of data on strain growth rates and substrate uptake rates or lack of growth on selected carbon sources. Comparison of these values is a key step in the basic evaluation of the model for correctness. Specifically, the iCGB21FR model was tested for completeness on multiple media for its ability to synthesize amino acids under both aerobic and anaerobic conditions. By varying the conditions of the cultivation medium, the limiting substrates in the biomass production reaction can be evaluated. This scenario is also suitable for assessing the ability to achieve the selected reactions under given cultivation medium conditions, i. e. to test the sufficiency of metabolites in the medium to potentially complete the targeted metabolic reactions

Scenario for evaluating the optimization of the space of feasible solutions

The previous scenario tested the implementation of targeted pathways from the point of substrate uptake to specific metabolic reactions. The next aspect of the study of such models is to assess the ability of the bacterium to operate under given conditions, i. e. the ability to synthesize a series of metabolites on a given substrate under the applied constraints in principle. Sampling methods for estimating the feasible solution space are helpful in this task. The solution in the “sampling” method is a vector of flux rates through all metabolic reactions that satisfies the balance conditions and user-applied constraints on the boundaries of the selec-ted reaction rates. In contrast to the flux balance analysis method, “sampling” generates a set of possible feasible solutions of the reaction system in the model without specifying target characteristics, which makes this method convenient for evaluating ways to optimize reactions (Herrmann et al., 2019).

**Fig. 4.**
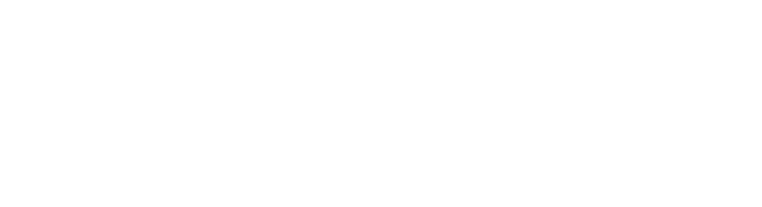
Comparison result of two variants of the iCGB21FR model: a baseline (“wild type”) model and a “knockout” model where a knockout of the periplasmic ATP synthase (atpB) gene is introduced. On the left – representation of lactate, valine and alanine excretion rate values; on the right – representation of the same values in one three-dimensional space (projections of 10,000 solution points on L-valine, D-alanine and L-lactate axes). Reaction flux rates in the model are expressed in mmol per gram of biomass dry weight per hour (mmol/(gDW × h)).

For a more accurate representation of the space of possible solutions, it is necessary to generate a sufficiently large number of samples with sizes of dozens/hundreds of thousands of points in the solution space (taking into account that each point in this space is described by hundreds or even thousands of numerical values of flow velocities). As a result, one can obtain a set of points in the solution space that can indicate the most frequent solutions under given conditions. The method uniformly selects points covering the solution space. By mapping the points to the coordinates of the target velocities, the expected distribution of values can be obtained. Thus, we do not get a specific distribution of fluxes on the metabolic map, but a series of solutions (a series of resultant fluxes/ cloud of points). Each point in this series of solutions can be mapped onto the rate axis of selected reactions of the metabolic network. This approach allows comparing flux distributions of both several models under the same conditions and one model under different conditions/constraints (Fig. 4).

In particular, a series of computational experiments on the effect of gene knockouts on metabolite excretion identified the atpB gene (KEGG cgb:cg1362), the synthesis product of which is involved in the ATP phosphorylation reaction (Fig. 4). Knockout of atpB provides potentially greater excretion of L-valine. Indirect evidence for the importance of this gene comes from the study (Jensen et al., 1993), which has shown that mutations in the ATP synthase operon in Escherichia coli can lead to a higher growth rate on glucose.

Running the calculations for 10 thousand solutions/points generates about 200 Mb of data in one run. Calculations and post-processing of such data are recommended to be performed on high-performance computational machines.

## Conclusion

The largest database of whole-genome models, BIGG (http:// bigg.ucsd.edu/models), has 108 models for 40 different or- ganisms. We found at least five whole-genome mathematical models on C. glutamicum, indicating a great interest in the object of study. The methodology of whole-genome modelling itself is still in the development stage and requires manual customization of tools for each new object. This gives a wide space for the development of mathematical and computational modelling techniques within the systems biologists/rational metabolic engineers’ community. Studies are now underway to incorporate transcriptomic and proteomic data into these types of models, leading to higher predictive power than simpler flux models.

Although C. glutamicum has been studied since 1956 (Kinoshita et al., 1957), gathering public information on strains of the bacterium is a challenge in itself. There are many strains for which the data is commercially available and may not be in the public domain. The development of computational pipelines will allow them to be applied to the metabolism of other strains in the future.

The proposed software module in the form of a series of computational protocols is configured for mass analysis of C. glutamicum strain models on cultivation on different nutrient media and under different environmental conditions (aerobic/anaerobic). The protocols are configured to run on a file-as-input/file-as-output basis, where the model, environment conditions, and model constraints are specified as separate files. Methods for visualization of simulation results have been set up, in particular for displaying data on a series of user-prepared metabolic maps. The specifics of algorithm execution require the use of high-performance computers and access to large amounts of data storage. The module is a part of the FluxMicrobiotech tool being developed at ICG SB RAS.

## Conflict of interest

The authors declare no conflict of interest.
